# Control of mitochondrial superoxide production by reverse electron transport at complex I

**DOI:** 10.1074/jbc.RA118.003647

**Published:** 2018-05-09

**Authors:** Ellen L. Robb, Andrew R. Hall, Tracy A. Prime, Simon Eaton, Marten Szibor, Carlo Viscomi, Andrew M. James, Michael P. Murphy

**Affiliations:** From the ‡ Medical Research Council Mitochondrial Biology Unit, Hills Road, University of Cambridge, Cambridge CB2 0XY, United Kingdom,; the § UCL Great Ormond Street Institute of Child Health, London WC1N 1EH, United Kingdom,; the ¶ Faculty of Medicine and Life Sciences, University of Tampere, Tampere FI-33014, Finland, and; the ‖ Max-Planck-Institute for Heart and Lung Research, Ludwigstrasse 43, 61231 Bad Nauheim, Germany

**Keywords:** mitochondria, mitochondrial membrane potential, redox signaling, reactive oxygen species (ROS), respiration, complex I, reverse electron transport, RET, coenzyme Q, superoxide

## Abstract

The generation of mitochondrial superoxide (O_2_^˙̄^) by reverse electron transport (RET) at complex I causes oxidative damage in pathologies such as ischemia reperfusion injury, but also provides the precursor to H_2_O_2_ production in physiological mitochondrial redox signaling. Here, we quantified the factors that determine mitochondrial O_2_^˙̄^ production by RET in isolated heart mitochondria. Measuring mitochondrial H_2_O_2_ production at a range of proton-motive force (Δp) values and for several coenzyme Q (CoQ) and NADH pool redox states obtained with the uncoupler *p*-trifluoromethoxyphenylhydrazone, we show that O_2_^˙̄^ production by RET responds to changes in O_2_ concentration, the magnitude of Δp, and the redox states of the CoQ and NADH pools. Moreover, we determined how expressing the alternative oxidase from the tunicate *Ciona intestinalis* to oxidize the CoQ pool affected RET-mediated O_2_^˙̄^ production at complex I, underscoring the importance of the CoQ pool for mitochondrial O_2_^˙̄^ production by RET. An analysis of O_2_^˙̄^ production at complex I as a function of the thermodynamic forces driving RET at complex I revealed that many molecules that affect mitochondrial reactive oxygen species production do so by altering the overall thermodynamic driving forces of RET, rather than by directly acting on complex I. These findings clarify the factors controlling RET-mediated mitochondrial O_2_^˙̄^ production in both pathological and physiological conditions. We conclude that O_2_^˙̄^ production by RET is highly responsive to small changes in Δp and the CoQ redox state, indicating that complex I RET represents a major mode of mitochondrial redox signaling.

## Introduction

**Figure 1.**
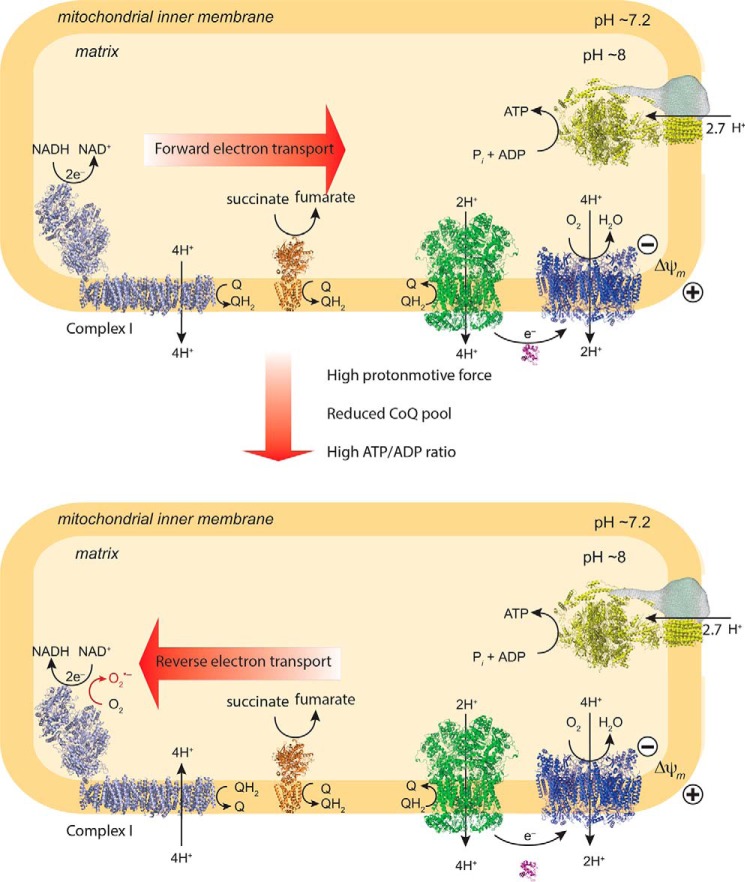
**Mitochondrial O_2_^˙̄^ production from complex I by RET.** The *top panel* shows conventional forward electron transport by mitochondria. The *bottom panel* shows mitochondrial O_2_^˙̄^ production by RET. This occurs when the Δp (a combination of the Δψ and the ΔpH) is high and the CoQ pool is reduced. *Q*, ubiquinone; *QH_2_*, ubiquinol.

Superoxide (O_2_^˙̄^)^3^ is the proximal reactive oxygen species (ROS) formed within mitochondria, with most O_2_^˙̄^ being very rapidly converted to H_2_O_2_ by manganese superoxide dismutase (MnSOD) within the matrix (1, 2). As well as contributing to oxidative damage, H_2_O_2_ acts as a redox signal, both within the mitochondria and in the cytosol (3–6). This mode of signal transduction arises via the reversible oxidation of protein thiols that pass on the modification to effector proteins as a redox relay (3–6). Whereas there are a number of potential mitochondrial sources of O_2_^˙̄^ (2, 7, 8), respiratory chain complex I is considered to be a major contributor (1). The production of O_2_^˙̄^ at complex I can be driven by reverse electron transport (RET) by a highly reduced coenzyme Q (CoQ) pool and a large proton-motive force (Δp), which together drive electrons backward through complex I and lead to a dramatic increase in O_2_^˙̄^ production (Fig. 1) (9). This process has been known since the 1960s (10) but was tacitly assumed to be an *in vitro* curiosity of no physiological relevance (1, 11, 12). However, there is now considerable evidence that RET at complex I is a physiological process that underlies mitochondrial redox signaling in a range of situations (11, 13) while also leading to pathological oxidative damage during ischemia–reperfusion injury (1, 14, 15).

A particularly intriguing aspect of RET is that it does not require damage to, or inhibition of, the respiratory chain (1, 9). As RET responds sensitively to physiological variables, O_2_^˙̄^ production by complex I can be modulated under physiological conditions (1, 9). Hence, there is considerable interest in understanding the mechanisms by which mitochondria regulate O_2_^˙̄^ production at complex I by RET as a physiological signaling process. Here, we quantified the factors that determine RET in isolated heart mitochondria. To do this, we measured H_2_O_2_ generation as a function of the membrane potential (Δψ) and of the reduction state of the CoQ and NADH pools. This was done by altering Δψ with the uncoupler carbonyl cyanide *p*-trifluoromethoxyphenylhydrazone (FCCP) and also oxidizing the CoQ pool by ectopic expression within heart mitochondria of the alternative oxidase (AOX) from *Ciona intestinalis* (16). Next, we assessed the dependence of RET O_2_^˙̄^ production on O_2_ concentration, [O_2_], and last, we determined how compounds known to alter mitochondrial ROS production affect RET. Together, these data provide a complete description of mitochondrial O_2_^˙̄^ production by RET and indicate how this process contributes to oxidative damage and redox signaling.

## Results

### Dependence of complex I O_2_^˙̄^ production by RET on Δp and CoQ redox state

To assess the factors that determine O_2_^˙̄^ production by RET at complex I, we used isolated heart mitochondria. H_2_O_2_ efflux from mitochondria is proportional to O_2_^˙̄^ production, assuming that O_2_^˙̄^ is converted to H_2_O_2_ by matrix MnSOD and that H_2_O_2_ degradation by peroxidases is similar across all conditions assessed (1). Whereas H_2_O_2_ movement across the plasma membrane is facilitated by aquaporins (17–19), these carriers are not present in mitochondria (20); hence, H_2_O_2_ efflux is probably unmediated, albeit facilitated by the large surface area of the inner membrane. Thus, measuring H_2_O_2_ efflux enables us to infer how O_2_^˙̄^ production by RET at complex I responds to mitochondrial state.

**Figure 2.**
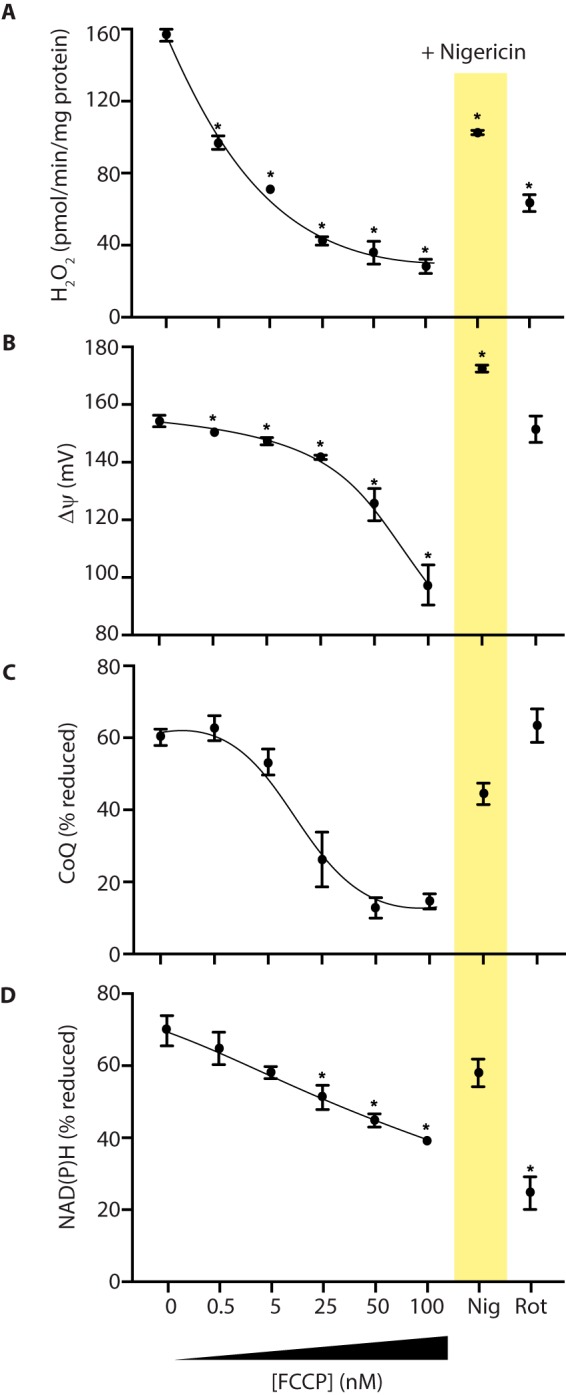
**Parallel measurement of mitochondrial H_2_O_2_ efflux, Δψ, and the redox status of the NAD(P)H and CoQ pools.** Rat heart mitochondria were incubated in the presence of potassium succinate. Where indicated, FCCP, nigericin (1 μm), or rotenone (5 μm) was added, and the indicated variables were measured. *A*, H_2_O_2_ efflux. *B*, Δψ. *C*, CoQ redox status. *D*, NAD(P)H redox status. Data are the mean ± S.E. (*error bars*) from 3–4 independent mitochondrial preparations. *, *p* < 0.05 compared with succinate-only conditions by ANOVA.

Three factors are critical in determining O_2_^˙̄^ production by RET: the Δp and the redox states of the mitochondrial CoQ and NADH pools (Fig. 1) (1). The O_2_ concentration is also likely to affect O_2_^˙̄^ production (1); however, these experiments were in air-saturated incubation medium ([O_2_] ∼200 μm); the dependence of O_2_^˙̄^ production on [O_2_] was determined later. The dependence of mitochondrial H_2_O_2_ production on these three variables was measured in parallel (Fig. 2). Δp was measured as Δψ from the distribution of the lipophilic methyltriphenylphosphonium (TPMP) cation (21) (Fig. 2*B*). Whereas this does not measure the pH gradient (ΔpH) across the inner membrane, ΔpH is a smaller (∼10–20%) component of Δp than Δψ and here is assumed to be invariant. The redox state of the CoQ pool was assessed by measuring its percentage reduction by reverse-phase HPLC (Fig. 2*C*). The redox state of the NADH pool was inferred from NAD(P)H fluorescence. Although this cannot distinguish between NADH and NADPH, the mitochondrial NADPH pool is thought to contribute less to changes in this variable (22); hence, this method gives a reasonable assessment of the NADH pool redox state (Fig. 2*D*).

The addition of succinate to heart mitochondria led to extensive H_2_O_2_ production (Fig. 2*A*), a large Δψ (Fig. 2*B*), and highly reduced CoQ (Fig. 2*C*) and NAD(P)H (Fig. 2*D*) pools. The addition of the complex I inhibitor rotenone decreased H_2_O_2_ production without affecting Δψ (Fig. 2*B*) or the CoQ pool (Fig. 2*C*), but there was oxidation of the NAD(P)H pool (Fig. 2*D*). These findings are consistent with H_2_O_2_ production originating from complex I by RET (1). To explore the dependence of RET on Δψ and the redox state of the CoQ and NAD(P)H pools, we measured these variables with increasing amounts of the uncoupler FCCP. The gradual decrease in H_2_O_2_ production that resulted (Fig. 2*A*) was associated with a decrease in Δψ (Fig. 2*B*) and oxidation of the CoQ (Fig. 2*C*) and NAD(P)H (Fig. 2*D*) pools.

The matrix pH has been suggested to alter RET directly at complex I, independently of its role as the ΔpH component of Δp (23, 24). Hence, we next used the K^+^/H^+^ exchanger nigericin to decrease the matrix pH from ∼7.7 to that of the incubation medium (pH 7.4), thereby abolishing the ΔpH component of Δp. Importantly, the magnitude of Δp will not change, due to a compensatory increase of ∼20 mV in Δψ (Fig. 2*B*). Nigericin resulted in an oxidation of the CoQ (Fig. 2*C*) and NAD(P)H pools (Fig. 2*D*) and a decrease in H_2_O_2_ efflux (Fig. 2*A*). Together, these data show a strong dependence of RET at complex I on the magnitude of Δp and on the CoQ and NAD(P)H redox states.

### Effect of the AOX on O_2_^˙̄^ production by RET

**Figure 3.**
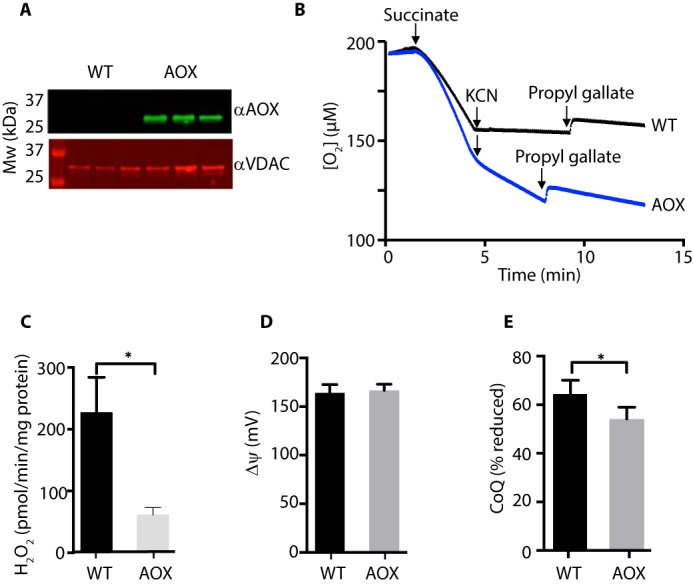
**Effect of AOX expression on mitochondrial H_2_O_2_ production.** *A*, AOX expression in mouse heart mitochondria. Heart mitochondria from AOX^+/−^ mice or WT littermate controls were analyzed by Western blotting for AOX using the mitochondrial outer membrane protein voltage-dependent anion channel as a loading control. Mitochondria from three separate AOX^+/−^ and WT mice were assessed. *B*, O_2_ consumption by mitochondria from AOX^+/−^ and WT mice. Mitochondria (200 μg of protein/ml) were incubated at 37 °C in an oxygen electrode and respiration was initiated by the addition of succinate (10 mm) followed by KCN (1 mm) and propyl gallate (50 μm). Traces are typical of experiments repeated with at least three independent mitochondrial preparations for each condition. *C*, H_2_O_2_ production by heart mitochondria from AOX^+/−^ and WT mice. Mitochondria (200 μg of protein/ml) were incubated with succinate (10 mm), and H_2_O_2_ production was assessed. *n* = 3 (WT) or 6 (AOX). *D*, Δψ of heart mitochondria from AOX and WT mice. Mitochondria (500 μg of protein/ml) were incubated at 37 °C for 5 min with succinate (10 mm), and Δψ was assessed. *n* = 4. *E*, CoQ redox state of heart mitochondria from AOX and WT mice. Mitochondria (1 mg of protein/ml) were incubated at 37 °C for 2 min with succinate (10 mm), and the CoQ redox state was assessed. *n* = 4. *, *p* < 0.05; *Error bars*, S.E.

To analyze the effects of CoQ pool redox state on O_2_^˙̄^ production by RET, independently of effects on Δp, we utilized mice expressing the AOX from *C. intestinalis* (16). AOX transfers electrons from CoQH_2_ directly to O_2_, bypassing complex IV, and thus acts as a safety valve to prevent the excessive reduction of the CoQ pool (25, 26). The AOX protein was present in heart mitochondria from AOX^+/−^ knock-in mice (Fig. 3*A*) and was catalytically active, as respiration in mitochondria from WT mice was inhibited by cyanide, whereas mitochondria from AOX mice continued to respire, but this residual respiration was sensitive to the AOX inhibitor *N*-propyl gallate (Fig. 3*B*). Mitochondrial H_2_O_2_ efflux during succinate oxidation was decreased by AOX expression as shown previously (16) (Fig. 3*C*). This was not due to a decrease in Δψ (Fig. 3*D*); however, the CoQ pool was more oxidized in mitochondria containing AOX (27) (Fig. 3*E*). These findings show that the expression of AOX affects O_2_^˙̄^ production by RET at complex I by oxidizing the CoQ pool and further support the importance of CoQ pool redox state in determining mitochondrial O_2_^˙̄^ production by RET.

### Effect of [O_2_] on O_2_^˙̄^ production by complex I RET

**Figure 4.**
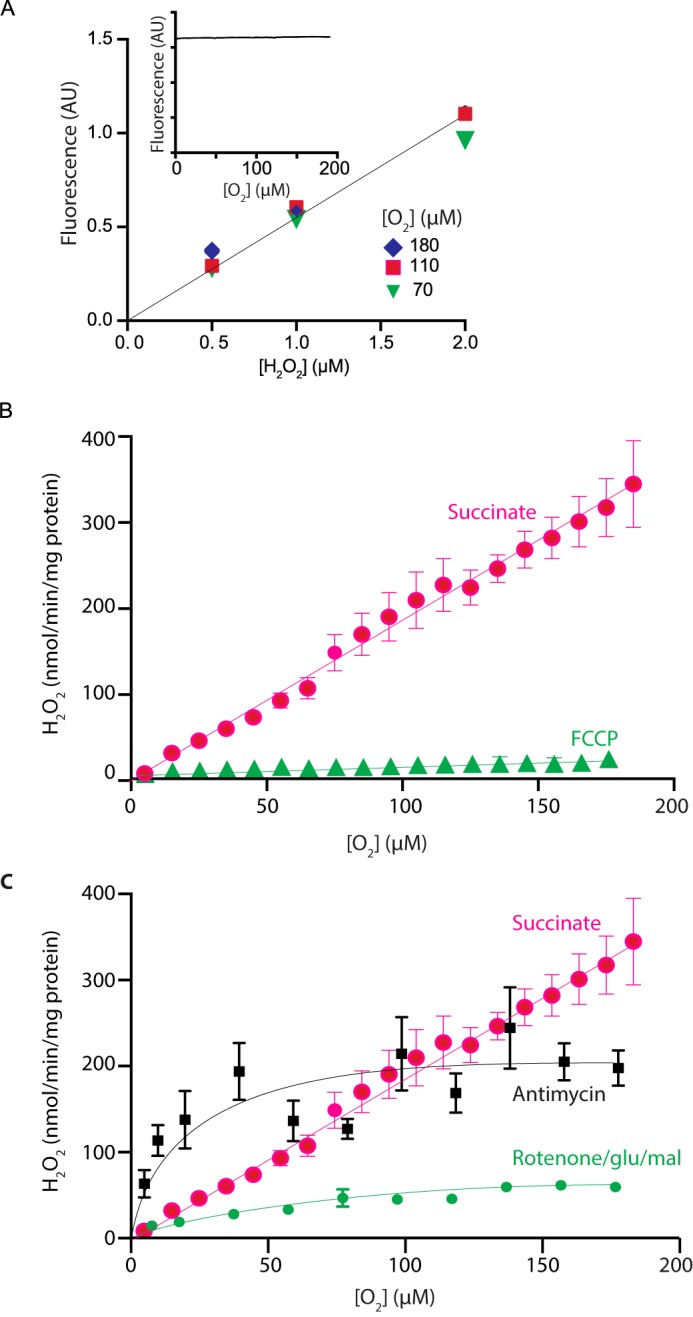
**Dependence on mitochondrial H_2_O_2_ generation on [O_2_].** *A*, conversion of Amplex Red to resorufin by HRP at different [O_2_]. The [O_2_] of KCl buffer containing Amplex Red, HRP, and SOD was decreased by bubbling with N_2_. Then known amounts H_2_O_2_ were injected, and fluorescence was measured. The *inset* shows the fluorescence of resorufin (25 μm), which was added to an incubation of mitochondria respiring on succinate in the presence of rotenone (5 μm), and resorufin fluorescence was measured as the [O_2_] decreased due to mitochondrial respiration. *B*, dependence on [O_2_] of mitochondrial H_2_O_2_ generation by RET. Rat heart mitochondria were incubated in an oxygen electrode, and H_2_O_2_ generation was measured at various [O_2_] set by bubbling with N_2_, and ROS production was measured over a dynamic range of O_2_ tensions. Where indicated, 500 nm FCCP was present. Data are the mean ± S.E. (*error bars*). *n* = 11. *C*, comparison of [O_2_] dependence of different modes of mitochondrial H_2_O_2_ generation. Mitochondria were assessed as in *B*, except that the respiratory substrate was glutamate/malate (5 mm each) in the presence of rotenone (4 μg/ml). *n* = 6, or mitochondria were respiring on succinate with antimycin (1 μm) present. Data are the mean ± S.E. (*n* = 5) and compared with the trace from *C*.

As the generation of O_2_^˙̄^ by mitochondria requires that O_2_ react with a protein-bound electron carrier, the rate of O_2_^˙̄^ production will probably depend on the [O_2_] (1). To assess this dependence, we measured mitochondrial H_2_O_2_ efflux at different [O_2_] (Fig. 4). As the apparent *K_m_* of cytochrome oxidase for O_2_ is very low (< 1 μm) (28), Δp and the redox state of the CoQ and NADH pools will not vary with [O_2_]. The assessment of H_2_O_2_ efflux at different [O_2_] relies on the conversion of Amplex Red to its fluorescent product resorufin catalyzed by horseradish peroxidase (HRP); hence, we first demonstrated that the fluorescence of resorufin (Fig. 4*A*, *inset*) and the conversion of Amplex Red to resorufin (Fig. 4*A*) were both independent of [O_2_]. We then measured mitochondrial H_2_O_2_ generation by RET and showed that it was proportional to [O_2_] and was abolished by excess FCCP (Fig. 4*B*). In contrast, production of H_2_O_2_ by NADH-linked substrates in the presence of rotenone (Fig. 4*C*) or from complex III upon inhibition of succinate respiration by antimycin (Fig. 4*C*) also showed increased H_2_O_2_ production with [O_2_], but in this case ROS production reached a plateau as [O_2_] increased. Thus, production of O_2_^˙̄^ by RET at complex I is proportional to [O_2_].

### Effects of therapeutic compounds on complex I RET

A number of potentially therapeutic compounds are thought to act, at least in part, by decreasing mitochondrial ROS production. Therefore, we set out to assess some of these compounds to determine whether they altered RET at complex I. The compounds tested were as follows: MitoQ, a mitochondria-targeted antioxidant based on ubiquinone (29); decylTPP, which contains the mitochondria-targeting triphenylphosphonium (TPP) cation and which is frequently used as a control compound to correct for nonspecific effects of MitoQ (21); SS31, a peptide composed of d-Arg-Dmt-Lys-Phe-NH_2_ (30) (where Dmt represents 2,6-dimethyltyrosine), whose therapeutic effects are thought to be due to its interactions with mitochondria; CN-POBS, an inhibitor of mitochondrial O_2_^˙̄^ production by RET at complex I (12); and the antidiabetic biguanides metformin and phenformin, which are known to interact with complex I (31, 32).

**Figure 5.**
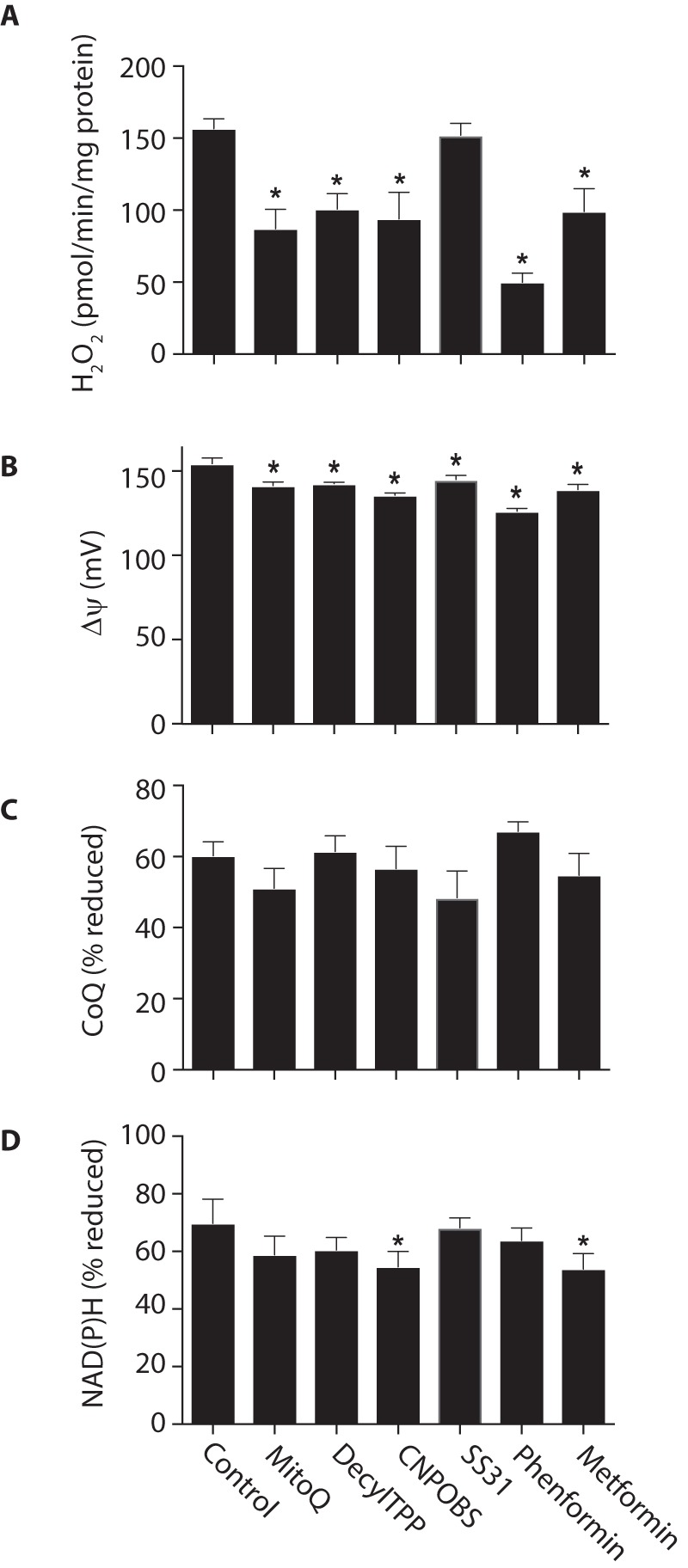
**Effects of compounds that interact with mitochondria on H_2_O_2_ generation by RET.** Rat heart mitochondria were incubated for 10 min in the presence of 10 mm succinate and 1 μm MitoQ, decylTPP, CN-POBS, SS31, phenformin, and metformin and assessed as in Fig. 2. Data are the mean ± S.E. (*error bars*), *n* = 4. *A*, H_2_O_2_ efflux. *B*, Δψ. *C*, CoQ redox status. *D*, NAD(P)H redox status. *, *p* < 0.05.

We first assessed whether these compounds affected H_2_O_2_ efflux by RET at complex I. All of the compounds, except SS31, decreased mitochondrial O_2_^˙̄^ production by RET to some extent (Fig. 5*A*). As RET at complex I is very sensitive to the magnitude of Δψ and redox state of the CoQ pool, these compounds may affect RET indirectly by altering these bioenergetic variables rather than by directly interacting with complex I to block RET-dependent O_2_^˙̄^ production. Consistent with this interpretation, all of the compounds decreased Δψ (Fig. 5*B*), and some affected the redox states of the CoQ and NAD(P)H pools (Fig. 5, *C* and *D*). Together, these findings suggest that the effects of these compounds on RET at complex I may be indirect due to effects on Δψ and perhaps on the redox states of the NAD(P)H and CoQ pools.

### Dependence of RET on Δψ and the redox state of the CoQ pool

**Figure 6.**
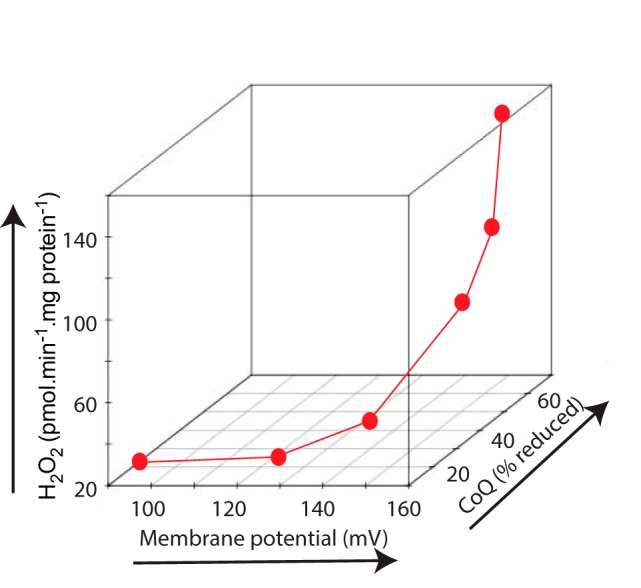
**Dependence of mitochondrial H_2_O_2_ production by RET upon Δψ and the redox state of the CoQ pool.** The data from Fig. 2 (*A–C*) are plotted together to show the relationship between mitochondrial H_2_O_2_ production, Δψ, and CoQ redox state.

Measuring H_2_O_2_ production by mitochondria in parallel with Δψ and the redox state of the CoQ pool indicated that O_2_^˙̄^ production by RET at complex I was very sensitive to both variables (Figs. 2 and 3). To illustrate the dependence of RET on Δψ and redox state of the CoQ pool, we replotted the data from Fig. 2 to show H_2_O_2_ production as a function of both Δψ and CoQ redox state (Fig. 6). This 3D plot makes evident the very steep dependence of H_2_O_2_ production on Δψ and on CoQ redox state, confirming the exquisite sensitivity of O_2_^˙̄^ production by RET at complex I to these two physiological variables.

### The thermodynamic driving force for RET at complex I

Whereas Fig. 6 shows clearly that decreasing Δψ and oxidizing the CoQ pool lowers RET, it does not provide a quantitative basis to allow us to infer whether the effects of nigericin shown in Fig. 2*A* and those of the various compounds shown in Fig. 5*A* are due to direct interactions with complex I itself or are indirect effects due to altering the driving forces of RET. Therefore, we next determined how O_2_^˙̄^ production during RET depends on the overall thermodynamic driving force across complex I.

The direction of electron flow at complex I is determined by the balance of thermodynamic driving forces across the complex (Fig. 1). During forward electron transfer at complex I, two electrons are passed from NADH to CoQ.

$$\begin{array}{c} \text{NADH}\;+\;\text{CoQ}↔{\text{NAD}}^{+}+{\text{CoQH}}_{2}\\ \text{Reaction}\;1 \end{array}$$

The driving force for the transfer of two electrons from NADH to CoQ is Δ*E_h_*.

(1) $$\Delta E_{h}=E_{h}\left(\frac{{\text{NAD}}^{+}}{\text{NADH}}\right)-E_{h}\left(\frac{\text{CoQ}}{{\text{CoQH}}_{2}}\right)$$

This driving force, 2Δ*E_h_*, is used to pump four protons across the mitochondrial inner membrane against the Δp. Hence, for forward electron movement to occur, the thermodynamic requirement is as follows.

(2) $$2\Delta E_{h}\;>\;4\Delta \text{p}$$

However, when the Δp is high and/or the Δ*E_h_* is decreased, RET can occur provided the following is true.

(3) $$2\Delta E_{h}\;<\;4\Delta \text{p}$$

We can thus calculate the thermodynamic driving force (Δ*G*) for RET, where *F* is the Faraday constant (33).

(4) $$\Delta G=2F\Delta E_{h}-\;4F\Delta \text{p}$$

Rearranging to express the thermodynamic driving force for RET as a positive number in V gives the following.

(5) $$-\Delta G/F=\;4\Delta \text{p}-2\Delta E_{h}$$

**Figure 7.**
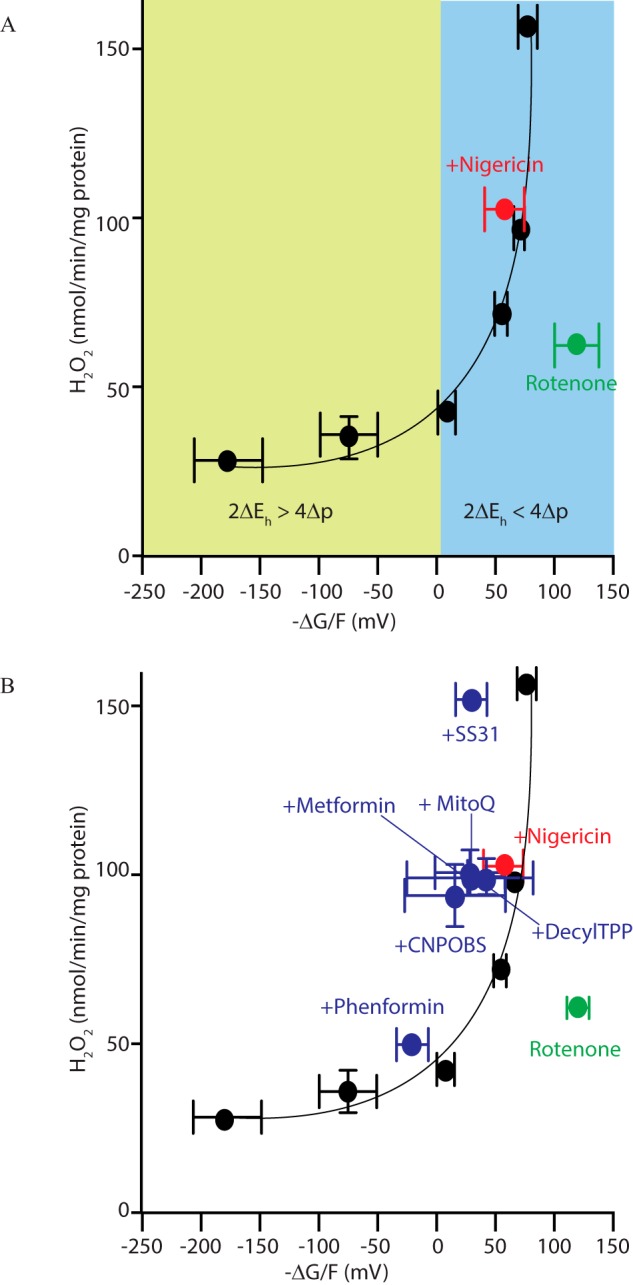
**Thermodynamic driving forces across complex I during RET.** *A*, dependence of mitochondrial H_2_O_2_ efflux on the thermodynamic driving force for RET across complex I. Data from Fig. 2 were used to calculate −Δ*G*/*F*, as this was varied by titration of the uncoupler FCCP (*black circles*) or in the presence of FCCP (*red circle*) or rotenone (*green circle*). Data are the mean ± S.E. (*error bars*) (*n* = 3–4). *B*, the effects of compounds that interact with mitochondria on H_2_O_2_ efflux and the thermodynamic driving force for RET. Data from Fig. 5 were used to calculate −Δ*G*/*F*, and this was plotted against mitochondrial H_2_O_2_ efflux (*blue circles*), along with the data from *A*.

From this, we can use the data from the FCCP titration in Fig. 2 to calculate −Δ*G*/*F*, the driving force for RET (see “Experimental procedures”). This analysis yields a plot of H_2_O_2_production by RET as a function of the thermodynamic driving force across complex I (Fig. 7*A*). This shows that when −Δ*G*/*F* < 0, there is a residual background level of ROS production, but as soon as the driving force for RET passes a threshold and −Δ*G*/*F* > 0, there is a dramatic and steep increase in O_2_^˙̄^ production by RET. This analysis confirms that the O_2_^˙̄^ production by RET requires a sufficient thermodynamic force to reverse electron transport at complex I and further shows the steep dependence of O_2_^˙̄^ production on this driving force.

A benefit of describing H_2_O_2_ production by RET at complex I as a function of its overall thermodynamic driving force is that it enables us to quantify whether compounds that affect O_2_^˙̄^ production by RET do so by altering the drivers of this process or by acting directly on complex I. A compound that only affects O_2_^˙̄^ production by RET indirectly through altering Δp and Δ*E_h_* would lie on the curve shown in Fig. 7*A*. In contrast, a compound that directly affected complex I independently of the thermodynamic drivers of RET, would lie below this curve. We first applied this analysis to nigericin, which decreases H_2_O_2_ production by RET (Fig. 2*A*) but which also led to a more oxidized CoQ pool (Fig. 2*C*) without affecting Δp. Carrying out this analysis, including accounting for changes in matrix pH on *E_h_* of the NADH and CoQ pools (see “Calculations”) indicated that the decrease in H_2_O_2_ production by nigericin was due to its effects on the CoQ pool redox state (Fig. 7*A*). In contrast, the decrease of H_2_O_2_ production by the complex I inhibitor rotenone (Fig. 2*A*) was not due to changes in the thermodynamic driving forces for RET, as these data lay below the trend line in Fig. 7*A*.

If the compounds that affect mitochondrial ROS production assessed in Fig. 5 decrease mitochondrial ROS production independently of the drivers of RET, then they should lie below the trend line shown in Fig. 7*A*, as was the case for rotenone. When the data from Fig. 5 were analyzed to show the effect of these compounds on the thermodynamic driving forces for RET, the results lay above the trend line, showing the dependence of H_2_O_2_ production on the thermodynamic driving forces across complex I (Fig. 7*B*). Hence, these data suggest that the effects of the compounds analyzed in Fig. 5 on ROS production by RET are more likely to be accounted for by their effects on Δp and/or Δ*E_h_* rather than due to specific inhibitory effects on complex I.

## Discussion

We investigated the dependence of O_2_^˙̄^ production by RET at complex I within isolated mitochondria on *E_h_* of the NAD(P)H and CoQ pools, Δp, matrix pH, and [O_2_]. This approach confirmed that O_2_^˙̄^ production by RET at complex I is favored by a high Δp and a reduced CoQ pool, with exquisite sensitivity to small changes in these two drivers (Fig. 6). This analysis was extended to show that O_2_^˙̄^ production by RET at complex I was also highly responsive to small changes in the overall thermodynamic driving force for RET across the complex (Fig. 7*A*).

Two sites have been proposed for O_2_^˙̄^ production by complex I during RET: the FMN of the complex I NADH-binding site (9, 34, 35) or the CoQ-binding site (36, 37). We favor the FMN site as the source of O_2_^˙̄^ production by complex I during RET. This is because the penetration of O_2_ to the CoQ site is difficult to envisage from the structure of complex I (38, 39). In addition, the generation of the negatively charged O_2_^˙̄^ from the CoQ site would require its thermodynamically unfavorable formation within the hydrophobic core of the membrane bilayer (38, 39). Furthermore, the FMN site is well established as a source of O_2_^˙̄^ production by rotenone-inhibited complex I (38, 39). During this process, NADH reduces the FMN to FMNH^−^, which is then readily accessed by O_2_ to form O_2_^˙̄^ (38, 39). In addition, any O_2_^˙̄^ formed at this site is released directly into the aqueous phase (9, 35, 40). The relationship between [O_2_] and RET was linear over the physiological [O_2_] range, consistent with O_2_^˙̄^ production being driven by the second-order reaction between O_2_ and FMNH^−^ on complex I when it is free from bound NAD^+^ or NADH (9, 35, 40).

During rotenone inhibition, the FMN/FMNH^−^ ratio is set by a rapid pre-equilibration with the matrix NAD^+^/NADH pool and thereby determines the rate of O_2_^˙̄^ production (9, 35). We favor FMNH^−^ as the donor of an electron to O_2_ for O_2_^˙̄^ production by complex I during RET. However, if this is the case then the greater O_2_^˙̄^ production during RET compared with rotenone-inhibited complex I has to be explained, because under both conditions, O_2_^˙̄^ production is determined by the FMN/FMNH^−^ ratio (9, 23, 24, 37). The most likely reason why RET-driven O_2_^˙̄^ production is greater than that upon rotenone inhibition is because the large thermodynamic driving force for electron movement backward through complex I during RET holds the FMN/FMNH^−^ ratio at a more negative *E_h_* than is possible by equilibrium with the NAD^+^/NADH pool (9). The more negative midpoint potential of the FMN/FMNH^−^ couple (*E_m_*_,7.5_ = −380 mV) (41) compared with the NAD^+^/NADH couple (*E_m_*_,7.5_ = −335 mV) is consistent with this hypothesis. Other factors that could contribute to the elevated O_2_^˙̄^ production during RET compared with rotenone inhibition include differential access of O_2_ to the FMNH^−^ due to alterations in the NADH and NAD^+^ binding or differences in the activity of the peroxidases that degrade H_2_O_2_ within the mitochondrial matrix. One further consideration is that during O_2_^˙̄^ production, FMNH^−^ donates one electron to O_2_ to form a semiquinone radical, FMN^•^, which is then thought to rapidly redistribute its unpaired electron throughout the iron–sulfur centers on complex I (40). However, the FMN^•^ radical also reacts very rapidly with O_2_ to form O_2_^˙̄^, so if electron redistribution were slowed during RET, the enhanced lifetime of FMN^•^ would also enhance O_2_^˙̄^ production (40).

**Figure 8.**
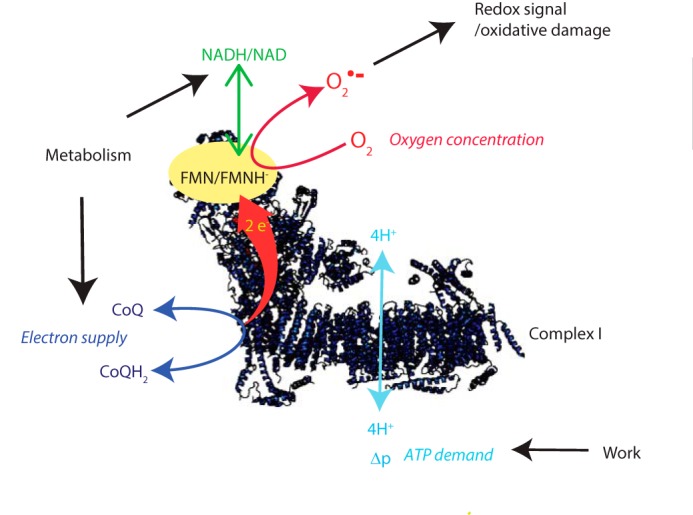
**Model of the factors that determine the production of O_2_^˙̄^ by RET at complex I.**

A further point to note is that the term RET is often interpreted as requiring NAD^+^ reduction at the FMN site of complex I. This is not the case, as O_2_^˙̄^ production by RET at complex I occurs when the NAD^+^/NADH pool is highly reduced and there is no net electron flow from complex I into this pool (*e.g.* Fig. 2*D*). Thus, we favor a model in which O_2_ reacts with a FMNH^−^ to generate O_2_^˙̄^, whereas the FMN still exchanges electrons with the matrix NAD^+^/NADH pool, although there is no net electron transfer (Fig. 8).

The generation of O_2_^˙̄^ by RET at complex I could be described as a function of the thermodynamic driving forces, Δp and Δ*E_h_*, across the complex. As well as illustrating the factors that drive RET, this analysis allowed us to integrate the effects of the forces driving RET. Applying this to the decrease in RET when the matrix pH decreased suggested that the lower rate of RET could be accounted for by the change in the Δ*E_h_* between the CoQ and NADH pools and may not be due to a direct effect of pH on complex I itself (24). Furthermore, this approach suggests that compounds such as MitoQ and metformin that affect mitochondrial ROS metabolism may do so indirectly, rather than by specific interactions with complex I. However, it is important to note that the calculation of −Δ*G*/*F* requires a number of assumptions and combines several technically challenging experimental measurements; hence, systematic errors will affect accuracy and precision. In addition, we have assumed that the CoQ pool interacts to the same extent with all complexes, and any effects of supercomplex formation on this were not considered (42). Nevertheless, our work does indicate novel approaches for determining how compounds impact mitochondrial ROS production and has implications for the interpretation of experiments using these compounds. For example, MitoQ is widely used *in vitro* and *in vivo*, where it acts as a chain-breaking antioxidant decreasing oxidative damage (29, 43). As the excessive accumulation of hydrophobic TPP compounds disrupts mitochondrial function (44, 45), controls with compounds with matched physicochemical properties are essential to correct for nonspecific effects. Thus, some “antioxidant” effects of lipophilic cations may be due to mild disruption of Δp that lowers mitochondrial O_2_^˙̄^ production by RET. Similarly, some of the beneficial effects of less hydrophobic cations, such as metformin *in vivo*, may be associated with limiting O_2_^˙̄^ production by RET, in addition to stimulating AMP-activated protein kinase by inhibiting complex I (46, 47).

Mitochondrial O_2_^˙̄^ production by RET was initially investigated *in vitro*, and at that time its relevance to *in vivo* physiology was not considered (10). Recently, mitochondrial ROS production by RET has been demonstrated in multiple situations *in vivo*. For example, during ischemia, high levels of succinate accumulate, and it is oxidized upon reperfusion to drive a burst of ROS via RET (14, 48). More generally, RET at complex I occurs as a redox-signaling pathway in inflammation (13), contributes to lifespan in flies (49), and is part of the oxygen-sensing mechanism of the carotid body (49). The linear dependence of ROS production by RET on O_2_ (Fig. 4), which has been shown previously by others (50–53), further adds to its appeal as a potential component of the carotid body O_2_ sensor (49). One possibility is that O_2_^˙̄^ production by RET at complex I accounts for most of the redox signaling from the mitochondrion to the rest of the cell (3–6). The appeal of RET as a mitochondrial redox signal is illustrated in Figs. 6 and 7, which show the tremendous sensitivity of RET to Δp and the redox status of the CoQ pool. The magnitude of Δp is directly linked to ATP demand, whereas the redox state of the CoQ pool reflects electron supply to the respiratory chain. Thus, RET provides a sensitive mechanism for the real-time feedback of the most critical aspects of mitochondrial status to the rest of the organelle and to the cell (Fig. 8).

In summary, we have provided a thermodynamic underpinning to O_2_^˙̄^ production by RET at complex I. This analysis highlights the potential for this mechanism to play a key role in mitochondrial redox signaling and demonstrates how to investigate the effects of RET on various physiological and pathological processes.

## Experimental procedures

### Mitochondria isolation

All procedures were performed in accordance with the UK Guide for the Care and Use of Laboratory Animals (PPL: 70/7538). Rat hearts were collected from 10–12-week-old female Wistar rats (Charles River). C57Bl/6 mice carrying a single copy of the *C. intestinalis* AOX gene in the *Rosa26* locus were generated as described (16). AOX mice and their WT littermate controls of both sexes were used at 8–12 weeks of age to prepare heart mitochondria. Rats were killed by stunning followed by cervical dislocation. Mice were killed by cervical dislocation only. Hearts were removed into ice-cold STE buffer (250 mm sucrose, 5 mm Tris-HCl (pH 7.4, KOH), and 1 mm K-EGTA, supplemented with 0.1% (w/v) fatty acid–free BSA. Heart mitochondria were isolated by homogenization and differential centrifugation (700 × *g* for 3 min; 3 × 5,500 × *g* for 10 min) at 4 °C. Mitochondrial protein content was determined using the bicinchoninic acid assay with BSA as a standard.

### Hydrogen peroxide efflux

H_2_O_2_ efflux from mitochondria was assayed using a plate reader fluorometer (SpectraMax GeminiXS, Molecular Devices; used at medium sensitivity). Resorufin (the product of Amplex Red oxidation) fluorescence was detected using λ_ex_ = 570 nm and λ_em_ = 585 nm. Mitochondria (2 mg protein/ml) were incubated with 2.5 μm Amplex Red (Invitrogen), 5 units/ml HRP in STE with 10 mm potassium succinate at 37 °C. H_2_O_2_ production rates were linear over 10 min but were measured from 0 to 2 min to facilitate comparison with other measurements. The H_2_O_2_ response was calibrated using freshly prepared H_2_O_2_ standards (ϵ_240_ = 43.5 m^−1^ cm^−1^) that were added sequentially to mitochondrial incubations lacking only succinate to generate a linear calibration curve. Mitochondria-targeted test compounds were added 30 s before measurement.

### Mitochondrial Δϵ

Mitochondrial Δϵ was measured by the uptake of radiolabeled [^3^H]TPMP as described (21). Mitochondria (2 mg protein/ml) were incubated at 37 °C with 10 mm succinate, 500 nm TPMP supplemented with [^3^H]TPMP (50 nCi/ml) with the test compounds for 2 min in 250 μl of medium in Eppendorf tubes. Mitochondria were pelleted by centrifugation (10,000 × *g* for 30 s). Supernatant (200 μl) was removed, and the pellets were dried with a rolled-up tissue and then solubilized in 40 μl of 20% (v/v) Triton X-100. Both the supernatant and pellets were then added to scintillant (Ultima-Gold liquid scintillant, PerkinElmer Life Sciences) and incubated for 1 h at room temperature and then vortexed, and [^3^H]TPMP content was assessed using a TriCarb LCS counter (PerkinElmer Life Sciences) counter with appropriate quench controls. To calculate the Δϵ, first the accumulation ratio was calculated assuming a mitochondrial matrix volume of 0.6 μl/mg protein, and the mitochondrial Δϵ was then calculated from the Nernst equation, assuming 40% binding of TPMP and that this was independent of mitochondrial Δϵ and consistent across all conditions (21).

### CoQ extraction and detection

Mitochondria (2 mg of protein/ml) were incubated in 500–700 μl of STE with 10 mm potassium succinate at 37 °C on a shaking heat block for 2 min. At the end of the incubation, mitochondria were rapidly pelleted by centrifugation (10,000 × *g* for 30 s), the supernatant was removed, and pellets were snap-frozen in a dry ice/ethanol bath and stored at −80 °C until analysis. Immediately before HPLC analysis, the pellets were homogenized in 0.5 ml of ice-cold, nitrogen-purged 1-propanol in an ice-cold glass-on-glass homogenizer, 100 μl of ice-cold H_2_O was added, and the samples were centrifuged (16,000 × *g* at 4 °C for 5 min). Supernatants (200 μl) were immediately analyzed by HPLC on a 150 × 4.6-mm, 3 μ Hypersil ODS column (Thermo). Solvent A was MeOH, 50 mm NaClO_4_; solvent B was EtOH, 50 mm NaClO_4_. The gradient was 60% to 50% A over 15 min at a flow rate of 0.8 ml/min at 45 °C. Identity was established by retention time compared with authentic standards at the absorbance maxima for CoQ and CoQH_2_ (260 and 290 nm, respectively). CoQ_9_ redox state was determined from the peak areas of ubiquinone and ubiquinol at 292.5 nm, the isosbestic point for oxidized and reduced CoQ_9_. The percentage reduction of the CoQ_9_ pool was calculated as area of the reduced peak divided by the sum of both peak areas. Control incubations under conditions designed to maximally oxidize and reduce the CoQ pool demonstrated that this approach accurately reported the CoQ redox state, as the percentage reduction increased to ∼80% in the presence of cyanide or anoxia, whereas inhibition of the respiratory chain with malonate decreased the percentage reduction to ∼20%.

### Redox state of mitochondrial NAD(P)H/NAD(P) pools

The redox state of the NAD(P)H/NAD(P)^+^ pool was determined by monitoring NAD(P)H fluorescence using a plate reader fluorimeter (SpectraMax GeminiXS; Molecular Devices) using λ_ex_ = 365 nm and λ_em_ = 450 nm. Mitochondria were incubated as described for measuring H_2_O_2_ efflux. The signal was calibrated by subtraction of background fluorescence (mitochondria with no additions), and maximal reduction of the pool was set by incubating mitochondria with 5 mm malate and 5 μm rotenone for 5 min.

### Combined respirometry and H_2_O_2_ production measurements

Combined respiration and H_2_O_2_ production by mitochondria was assessed using an Oxygraph2K (O2K) respirometer (Oroboros, Innsbruck, Austria) with a fluorescence LED module attachment. To assess the effect of O_2_ concentration on H_2_O_2_ production, mitochondria (200–250 μg of protein/ml) were suspended in 2 ml of KCl buffer (120 mm KCl, 10 mm Hepes, 1 mm EGTA, pH 7.2) supplemented with 50 units/ml SOD, 4 units/ml HRP, 0.2 mg/ml fatty acid–free BSA, 25 μm Amplex Red with stirring at 37 °C. Respiration was initiated by the addition of either 5 mm glutamate and 5 mm malate; 10 mm succinate; or 10 mm succinate with 5 μm rotenone. Where indicated, incubations were supplemented at the start with 5 μm rotenone, 500 nm FCCP, 1 μm antimycin A, or 1 μm nigericin. The concentration of O_2_ was adjusted by bubbling the buffer with N_2_. Amplex Red fluorescence was measured via the O2K fluorometer, and the corresponding voltage changes were calibrated via titration of known amounts of H_2_O_2_ (500 nm to 5 μm) in the presence of mitochondria, SOD, fatty acid–free BSA, HRP, and Amplex Red.

### Western blotting

Mitochondrial pellets (∼250 μg of protein) were solubilized on ice in 50 μl of lysis buffer (100 mm Tris, 300 mm NaCl, 0.05% Nonidet P-40, pH 7.4, supplemented with protease and phosphatase inhibitors (Roche Applied Science). Protein was then quantified by the BCA assay and diluted in 4× loading buffer (Invitrogen), and 10 μg of protein was separated by SDS-PAGE on a 10% gel and transferred to polyvinylidene difluoride membrane. Membranes were incubated with a 1:20,000 dilution of rabbit serum raised against two AOX peptides (FKIETNDSTDEPNIEVENFPC and CVNHDLGSRKPDEQNPYPPGQ (49)) and a mouse monoclonal antibody against the voltage-dependent anion channel (1:1,000; Abcam ab14734)) and visualized using a LI-COR Odyssey flatbed scanner with anti-mouse and anti-rabbit secondary antibodies conjugated to IRDye 680RD and IRDye 800CW, respectively.

### Calculations

This section describes calculations required to determine the thermodynamic driving force for RET from the data in Figs. 2 and 5 to generate the graphs shown in Fig. 7. The thermodynamic driving force for RET is derived from Equation 5. In mV, it is as follows.

(6) Δp=Δψ−61.5ΔpH

In the presence of nigericin, Δp is unchanged, whereas ΔpH = 0. Hence, ΔpH = Δψ (+nigericin) − Δψ (−nigericin). From Fig. 2*C*, 61.5ΔpH = −18.1 mV and the matrix pH = 7.7.

(7) Δp=Δψ+18.1

As the matrix pH is 7.7, *E_h_* for the NAD^+^/NADH couple in mV is as follows.

(8) $$E_{h}\left({\text{NAD}}^{+}/\text{NADH}\right)=-341+30.5\log_{10}\left({\text{NAD}}^{+}/\text{NADH}\right)$$

In the presence of nigericin, the matrix pH will be 7.4, and under those conditions, the following will be true.

(9) $$E_{h}\left({\text{NAD}}^{+}/\text{NADH}\right)=-332+30.5\log_{10}\left({\text{NAD}}^{+}/\text{NADH}\right)$$

As the matrix pH is 7.7, *E_h_* for the CoQ/CoQH_2_ couple in mV is as follows.

(10) $$E_{h}\left(\text{CoQ}/{\text{CoQH}}_{2}\right)=-38+30.5\log_{10}\left(\text{CoQ}/{\text{CoQH}}_{2}\right)$$

In the presence of nigericin, the matrix pH will be 7.4, and under those conditions, the following will be true.

(11) $$E_{h}\left(\text{CoQ}/{\text{CoQH}}_{2}\right)=-20+30.5\log_{10}\left(\text{CoQ}/{\text{CoQH}}_{2}\right)$$

### Statistical analysis and experimental design

Data were expressed as mean ± S.E., with *p* values calculated using a two-tailed Student's *t* test for pairwise comparisons whereas one-way analysis of variance (ANOVA) followed by Tukey's post hoc test was used for multiple comparisons. Statistical analyses were performed using GraphPad Prism version 7 software.

## Author contributions

E. L. R. and A. R. H. designed and carried out the experiments, with assistance from T. A. P. S. E. carried out the CoQ assays. M. S. and C. V. provided the AOX mice. A. M. J. provided advice and helped with data interpretation. M. P. M. directed the project and wrote the manuscript, with assistance from all other authors.

## Footnotes

3 The abbreviations used are:

O_2_^˙̄^: superoxide
ROS: reactive oxygen species
SOD: superoxide dismutase
RET: reverse electron transport
CoQ: coenzyme Q
Δp: proton-motive force
Δψ: membrane potential
FCCP: carbonyl cyanide *p*-trifluoromethoxyphenylhydrazone
AOX: alternative oxidase
TPMP: methyltriphenylphosphonium
HRP: horseradish peroxidase
TPP: triphenylphosphonium
ANOVA: analysis of variance
CN-POBS: *N*-cyclohexyl-4-(4-nitrophenoxy)benzenesulfonamide.
